# The Cellular Immunological Responses and Developmental Differences between Two Hosts Parasitized by *Asecodes hispinarum*

**DOI:** 10.3390/life12122025

**Published:** 2022-12-04

**Authors:** Zhiming Chen, Tingting Fu, Lang Fu, Bin Liu, Yaping Lin, Baozhen Tang, Youming Hou

**Affiliations:** 1 State Key Laboratory of Ecological Pest Control for Fujian and Taiwan Crops, Fujian Agriculture and Forestry University, Fuzhou 350002, China; 2 Integrated Technical Service Center of Rongcheng Customs, Fuzhou 350015, China; 3 Fujian Province Key Laboratory of Insect Ecology, Department of Plant Protection, Fujian Agriculture and Forestry University, Fuzhou 350002, China; 4 Key Lab of Biopesticide and Chemical Biology, Ministerial and Provincial Joint Innovation Centre for Safety Production of Cross-Strait Crops, Fujian Agriculture and Forestry University, Fuzhou 350002, China

**Keywords:** *Asecodes hispinarum*, *Brontispa longissima*, *Octodonta nipae*, developmental interactions, cellular immune, hemocytes

## Abstract

This study aims to investigate the developmental interactions of *Asecodes hispinarum* Bouček on *Brontispa longissima* Gestro and *Octodonta nipae* Maulik, as well as the cellular immune responses of *B. longissima* and *O. nipae* larvae in response to parasitism by *A. hispinarum*, with the hope of determining the reason for the difference in larval breeding of *A. hispinarum* in *B. longissima* and *O. nipae*. The effects of parasitism by *A. hispinarum* on the larval development, hemocyte count, and proportion of the hemocyte composition of the two hosts were carried out through selective assay and non-selective assay using statistical analysis and anatomical imaging. There was no significant difference in parasitic selection for *A. hispinarum* on the larvae of these two beetles; however, more eggs were laid to *B. longissima* larvae than to *O. nipae* larvae after parasitism by *A. hispinarum*. The eggs of *A. hispinarum* were able to grow and develop normally inside the larvae of *B. longissima*, and the parasitism caused the larvae of *B. longissima* become rigid within 7 d, with a high larval mortality rate of 98.88%. In contrast, the eggs of *A. hispinarum* were not able to develop normally inside the *O. nipae* larvae, with a high encapsulation rate of 99.05%. In addition, the parasitism by *A. hispinarum* caused a 15.31% mortality rate in *O. nipae* larvae and prolonged the larval stage by 5 d and the pupal stage by 1 d. The number of hemocytes during the 12, 24, 48, 72, and 96 h of the four instars from *O. nipae* larvae was 6.08 times higher than from *B. longissima* larvae of the same age. After 24 h of being parasitized by *A. hispinarum*, the total number of hemocytes and granulocyte proportion of *B. longissima* larvae increased significantly. However, the total number of hemocytes and plasmatocyte proportion of *O. nipae* increased significantly after 24, 72, and 96 h, and the proportion of granulocytes increased significantly after 12 h post-parasitism. The results in the present study indicated that *A. hispinarum* was unable to successfully reproduce offspring in *O. nipae*, but its spawning behavior had an adverse effect on the larval development of its host. In addition, the adequate number of hemocytes and more pronounced changes in the hemocyte count and hemocyte composition ratio in the larvae after parasitization may be important factors for the successful encapsulation in *O. nipae* larvae.

## 1. Introduction

The coconut leaf beetle, *Brontispa longissima* Gestro (Coleoptera: Chrysomelidae), is thought to be native to Indonesia and Papua New Guinea. The nipa palm hispid beetle, *Octodonta nipae* Maulik (Coleoptera: Chrysomelidae), is native to Malaysia. These species have invaded China with the international trade in seedlings and are currently wreaking havoc in southern China [1,2,3,4]. The two beetles attack young leaf fronds of different palm plants, leaving behind small brown spots that run parallel to veins and can even kill the entire plant [3,5]. As a result, the decorative palm sector in China suffers large palm losses every year [3,6].

For these two beetles, chemical and biological control are currently significant control methods. The high stems of palm plants combined with the characteristics of these two beetles, such as feeding and lodging in the tightly furled fronds and trunk fibers, render typical chemical control ineffectual [7]. There have been numerous successful reports of using local natural enemies or introducing natural enemies to manage these two invasive beetles, which is why many nations and areas focus on this approach [8].

Different insects can be parasitized by parasitic wasps; however, not all parasitized insects can produce parasitoid offspring. Adaptive hosts are insects that parasitic wasps may effectively parasitize and breed in, whereas non-adaptive hosts are insects that are incapable of producing progeny [9,10,11,12]. After being parasitized, insect immune systems react right away [13,14]. There are two types of immunological responses in insects: cellular immunity and humoral immunity. Cellular immunity uses hemocytes to enclose, phagocytose, and nodulate foreign compounds [15,16]. The encapsulation in host cellular immunity is the initial immunological response that the parasitic wasp encounters [12,17]. The encapsulation response requires a sufficient number of immunological hemocytes [18,19]. The type, quantity, and composition of hemocytes are significant indicators in depicting the strength of host cell immunity [20,21,22,23].

A gregarious and koinobiont endoparasitoid native to Western Samoa and Papua New Guinea [24], *Asecodes hispinarum* Bouček (Hymenoptera: Eulophidae) demonstrates an enhanced potential in the biocontrol of *B. longissima* larvae [8,25,26,27]. Understanding how *A. hispinarum* manipulates the physiology and biochemistry of *B. longissima* larvae to generate an environment favorable for the development of its progeny is important for developing an efficient pest-management approach [23,28,29,30,31].

We observed that *O. nipae* larvae were also parasitized, but the eggs of *A. hispinarum* that were encapsulated cannot develop normally. Invasive pests can be managed with the help of parasitic wasps in a long-lasting and environmentally responsible manner. The parasitic wasp and the host engage in interaction, and the parasitic aspect of the parasitoid engages the immune system of the host. The focus of most research has always been on the aforementioned biological phenomena and mechanisms [32,33,34,35].

This study compared the interactions between parasitoid-adaptive host and parasitoid-non-adaptive host from the perspectives of biological phenomenon and the function of immune hemocytes in order to better understand the developmental and immune interactions between parasitoids and their hosts. *A. hispinarum*, its adaptive host *B. longissima*, and its non-adaptive host *O. nipae* were used as research objects.

## 2. Materials and Methods

### 2.1. Experimental Insects

*B. longissima* and *O. nipae* samples were obtained in July 2017 from the diseased *Phoenix canariensis* Hort.ex Chabaud host tree in Xiamen City (Fujian Province) (24.52° N, 118.18° E) and were introduced alive with a natural food source into the laboratory (fresh leaves of *P. canariensis*). The F4 laboratory generations were used for the experiment. The Chinese Academy of Tropical Agricultural Sciences provided the *A. hispinarum*. All insects were kept at (25 ± 1) °C, (70 ± 5)% RH, and 12 h:12 h photoperiod (light:dark).

### 2.2. A. hispinarum Parasite Selection on B. longissima and O. nipae Larvae

#### 2.2.1. Non-Selective Parasitism

In a plastic box measuring 15 cm long, 6 cm wide, and 4.5 cm high, 30 *B. longissima* larvae in their fourth instar were chosen and fed on fresh *P. canariensis* leaves. After 0.5 days of rearing, the larvae were parasitized with 30 mated one-day-old females of *A. hispinarum,* and the parasitic wasps were fed cotton dipped in 10% sucrose in plastic boxes. After 24 h, the parasitized larvae were dissected. The average parasitism rate and fecundity per female were obtained by counting the number of parasitized larvae and eggs per larva. The experiment was repeated in 40 groups, but only 10 groups had the *A. hispinarum* eggs counted.

*O. nipae* used the same experimental procedure as *B. longissima*.

#### 2.2.2. Selective Parasitism

In the same plastic box, thirty 4th instar larvae of *B. longissima* and thirty 4th instar larvae of *O. nipae* were chosen and fed on fresh *P. canariensis* leaves, respectively. Sixty mated one-day-old female *A. hispinarum* were introduced to the parasitoid wasps after 0.5 days of upbringing, and the parasitic wasps were fed cotton dipped in 10% sucrose in plastic containers. After 24 h, the parasitized larvae were dissected. The average parasitism rate and fecundity per female were obtained by counting the number of parasitized larvae and eggs per larva. The experiment was repeated in 40 groups, but only 10 groups had the *A. hispinarum* eggs counted.

### 2.3. Developmental Interaction of A. hispinarum with B. longissima and O. nipae Larvae

#### 2.3.1. The Development of *A. hispinarum* Eggs in the Body of *B. longissima* and *O. nipae* Larvae

*B. longissima* larvae in their fourth instar were selected, placed in separate 9 cm petri dishes, and parasitized by fifteen *A. hispinarum* mating females in each dish. We dissected *B. longissima* larvae that were infected by *A. hispinarum*, using a differential interference microscope. We also observed their progress and captured images at 12, 24, 48, 72, and 96 h after the two insects lay their eggs. Each time point involved the repetition of three groups. The same experimental protocol as *B. longissima* was followed by *O. nipae*.

The eggs were stained with rhodamine phalloidin and DAPI and photographed using a differential interference microscope within 24 h. These are the precise steps: The eggs were transferred into a slide coated with poly-L-lysine after 10 μL of PBS was poured upon it. Remove the liquid, absorb 10 μL of PBS rinse, repeat three times, add 10 μL of 4% paraformaldehyde, and then place the wet box in a fixed-temperature environment for 15 min. Rinse three times with 10 μL of PBS, then add 10 μL of 0.1% Triton X-100 and incubate for five minutes in a moist box. Following three rinses with 10 μL of PBS, 10 μL of 1% BSA was added to the wet box for one hour. After three rinses with 10 μL PBS, 10 μL Rhodamine phalloidin and 5 μL (1 μg/μL) DAPI were added and stained in a wet box for 45 min. Rinse three times with 10 μL PBS, then add another 10 μL PBS, cover with a coverslip, and photograph with a differential interference microscope.

#### 2.3.2. Effects of *A. hispinarum* Parasitism on *B. longissima* and *O. nipae* Larvae Development

*B. longissima* and *O. nipae* larvae that were parasitized by *A. hispinarum* were observed and captured under a stereo microscope; 30 *B. longissima* 4th instar larvae and 30 *O. nipae* 4th instar larvae were chosen and placed in separate 9 cm petri dishes. One-day-old, mated female *A. hispinarum* parasitized each larva one at a time. *A. hispinarum* was removed 24 h later. The parasitized larvae were constantly fed until they either pupated or became stiff. To count the number of larval deaths, *B. longissima* and *O. nipae* fourth instar larvae that were not parasitized throughout the same period were utilized as controls. The experiment was carried out in 30 groups. Ten fourth-instar *O. nipae* larvae were chosen, and 30 mated female *A. hispinarum* were inserted to perform one-for-one parasitism. The larvae were used as a control, and the pupal and fourth instar larvae of the palmar anise were counted. The experiment was carried out in thirty groups.

### 2.4. Comparison of the Encapsulation Rates of A. hispinarum Eggs with B. longissima and O. nipae Larvae

Thirty *B. longissima* 4th instar larvae and thirty *O. nipae* 4th instar larvae were dissected, and the total number of eggs in each larva and the number of eggs encapsulated were counted.

### 2.5. The Effect of A. hispinarum Parasitism on the Number and Proportion of B. longissima and O. nipae Larvae Hemocytes

The hemolymph of *B. longissima* fourth instar larvae was collected at 12, 24, 48, 72, and 96 h after parasitization, and the hemolymph of non-parasitic *B. longissima* larvae was used as a control at each time. The hemocytes were counted with a blood cell counter (25 × 16) under an optical microscope, including the number of cells and blood cell types; this was repeated five times. At each time point, 30 larvae were taken as replicates, and the experimental method was the same for *O. nipae* larvae.

### 2.6. Satistical Analysis

The mortality of of *B. longissima* and *O. nipae* larvae under non-parasitization and parasitization, the selective parasitism rate of *A. hispinarum* to *B. longissima* and *O. nipae* larvae, and the egg encapsulation rate of these two hosts after parasitization were analyzed using the Chi-square test. The fecundity of *B. longissima* and *O. nipae* larvae after parasitization, the duration of the larvae’s fourth instar and pupal stage, the hemocyte count, and the proportion of different hemocyte types under parasitization and non-parasitization were compared using an independent sample t-test. The changes in the proportion of hemocyte types at different time points post-parasitization and non-parasitization were analyzed using ANOVA. All statistical analysis mentioned above were performed on SPSS 21, and the graphs were drawn using GraphPad Prism 7.

## 3. Results

### 3.1. Parasitic Selection of A. hispinarum to B. longissima and O. nipae Larvae

There was no difference in the parasitism selection of *A. hispinarum* to *B. longissima* and *O. nipae* larvae in the non-selective parasitism experiment (*p* = 0.859), although there was a significant difference in the number of eggs laid (*p* < 0.001). The number of eggs produced in the *B. longissima* larvae was more than that in the *O. nipae* larvae (Table 1).

There was no difference in the parasitism selection of *A. hispinarum* to *B. longissima* and *O. nipae* larvae in the selective parasitism experiment (*p* = 0.568). In terms of oviposition, there was a significant difference in the quantity of eggs laid (*p* < 0.001). The quantity of eggs deposited in the *B. longissima* larvae was more than that in the *O. nipae* larvae (Table 1).

### 3.2. The Developmental Interaction of A. hispinarum with B. longissima and O. nipae Larvae

#### 3.2.1. Development of *A. hispinarum* Egg in *B. longissima* and *O. nipae* Larvae

**Figure 1:**
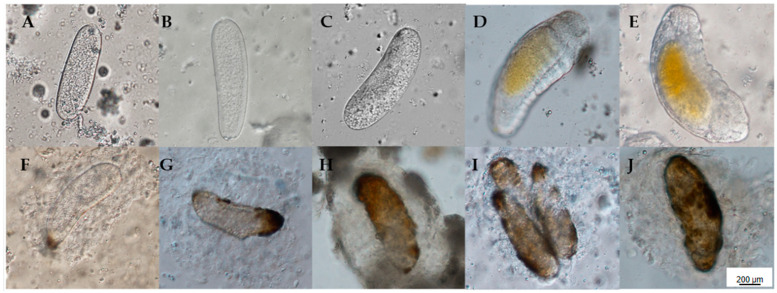
Development of *A. hispinarum* eggs in *B. longissima* and *O. nipae* larvae. (**A**–**E**), *A. hispinarum* eggs in the fourth instar larvae of *B. longissima* parasitized by *A. hispinarum* after 12/24/48/72/96 h; (**F**–**J**), *A. hispinarum* eggs in the fourth instar larvae of *O. nipae* parasitized by *A. hispinarum* after 12/24/48/72/96 h. Scale bar = 200 μm.

*A. hispinarum* eggs laid in *B. longissima* larvae can grow normally and mature into larvae 72 h later, according to ongoing observations of eggs laid in *B. longissima* and *O. nipae* larvae (Figure 1A–E). The eggs were encapsulated in *O. nipae* larvae from 12 h on, but as the encapsulation grew stronger over time, the eggs were unable to develop normally and eventually died (Figure 1F–J).

**Figure 2:**
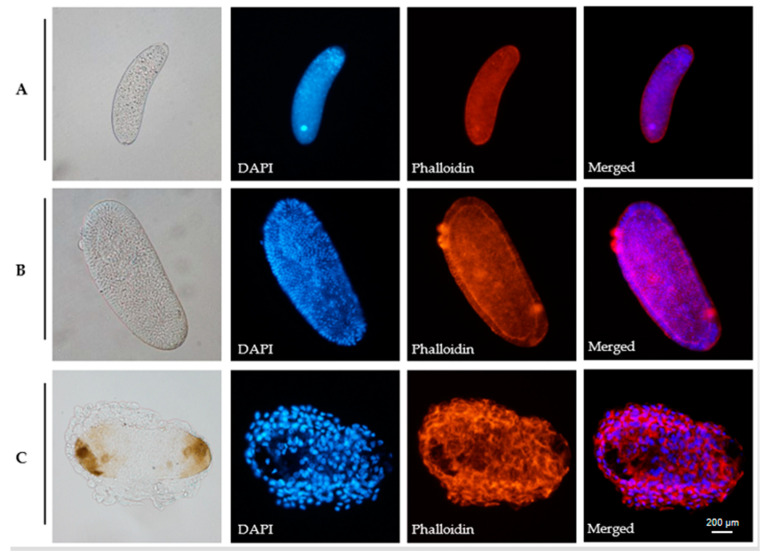
Encapsulation of *A. hispinarum* eggs in different sites. (**A**) The fallopian tube of *A. hispinarum*; (**B**) The fourth instar larvae of *B. longissima* parasitized by *A. hispinarum* after 24 h; (**C**) The fourth instar larvae of *O. nipae* parasitized by *A. hispinarum* after 24 h. Nuclei are stained with DAPI (blue) and the hemocytes stained with Phalloidin (red). Merged is the merged picture of blue and red channels. Scale bar = 200 μm.

It can be seen that the eggs could develop normally in the *B. longissima* larvae (Figure 2B), and they formed a blastoderm within 24 h, after staining to observe the eggs in fallopian tubes of *A. hispinarum* and those laid in *B. longissima* and *O. nipae* larvae 24 h later (Figure 2A) (Figure 2B Phalloidin). The eggs laid in the larvae of *O. nipae* can also develop, but they asphyxiate until they die, because the egg surface is covered in thick hemocytes, in contrast to the eggs in the fallopian tubes of *A. hispinarum*. (Figure 2C).

#### 3.2.2. The Effect of *A. hispinarum* Parasitism on the Development of *B. longissima* and *O. nipae* Larvae

**Figure 3:**
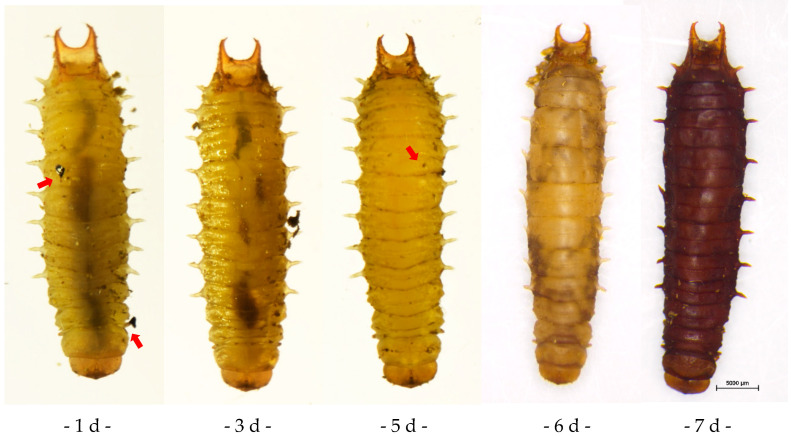
The growth and development of *B. longissima* larvae parasitized by *A. hispinarum.* (The red arrow is where the parasite wasp spawned. Scale bar = 5000 μm.).

**Figure 4:**
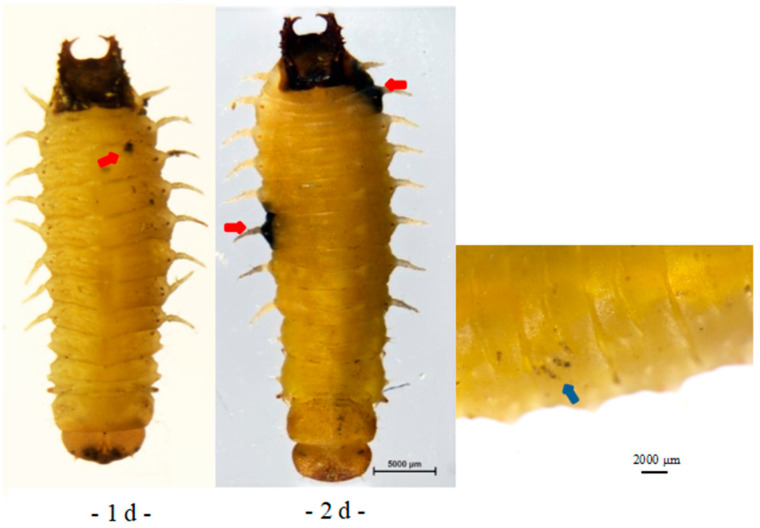
The growth and development of *O. nipae* larvae parasitized by *A. hispinarum.* (The red arrow is where the parasite wasp spawned. The blue arrow is the egg of *A. hispinarum*. Scale bar = 5000 μm/2000 μm.).

*A. hispinarum* parasitized *B. longissima* larva, leaving behind black spots on the body surface where the parasites lay their eggs. The parasitized *B. longissima* larva gradually turned black over a period of seven days (see Figure 3: the red arrow is where the parasite wasp spawned). *O. nipae* larva showed localized blackening around the spawning position after being parasitized by *A. hispinarum* (Figure 4, red arrow), and the eggs enclosed in the larva could be seen through the body surface (Figure 4, blue arrow). The parasitized larvae can still be observed with the encapsulated eggs until they become adults after extended observation.

*A. hispinarum* parasitized *B. longissima, O. nipae* larvae were raised, and the uninfected *B. longissima* and *O. nipae* larvae served as the control to measure the mortality of the two insects (Table 2). When *B. longissima* larvae were parasitized by *A. hispinarum*, their death rate was 98.88% ± 0.33%, which was substantially greater than the unparasitized rate of (1.11% ± 0.33%) (*χ*^2^ = 1716.890, *df* = 1, *p* < 0.001). *O. nipae* larvae infected with *A. hispinarum* had a mortality rate of 15.31% ± 1.62% (Table 2), which was significantly greater than the rate of uninfected larvae (1.44% ± 0.35%) (*χ*^2^ = 112.768, *df* = 1, *p* < 0.001). The death rates of unparasitized *B. longissima* and *O. nipae* larvae are identical (*χ*^2^ = 0.184, *df* = 1, *p* = 0.668). The death rate of *B. longissima* larvae was considerably higher than that of *O. nipae* larvae following parasitism by *A. hispinarum* (*χ*^2^ = 1275.909, *df* = 1, merged DAPI, merged phalloidin DAPIDAPIABC8 *p* < 0.001).

The statistics of the larval and pupal stages of *O. nipae* larvae surviving after being parasitized by *A. hispinarum* (Table 3) showed that the fourth instar larval stage is 12.55 ± 0.09 d, which was 5 d longer than the unparasitized one (7.17 ± 0.08 d); the pupal stage of parasitized was 10.64 ± 0.08 d, which was 1 d longer than the unparasitized one (8.96 ± 0.06 d).

### 3.3. Comparison of Encapsulation Rate of B. longissima and O. nipae Larvae to A. hispinarum Eggs

**Figure 5:**
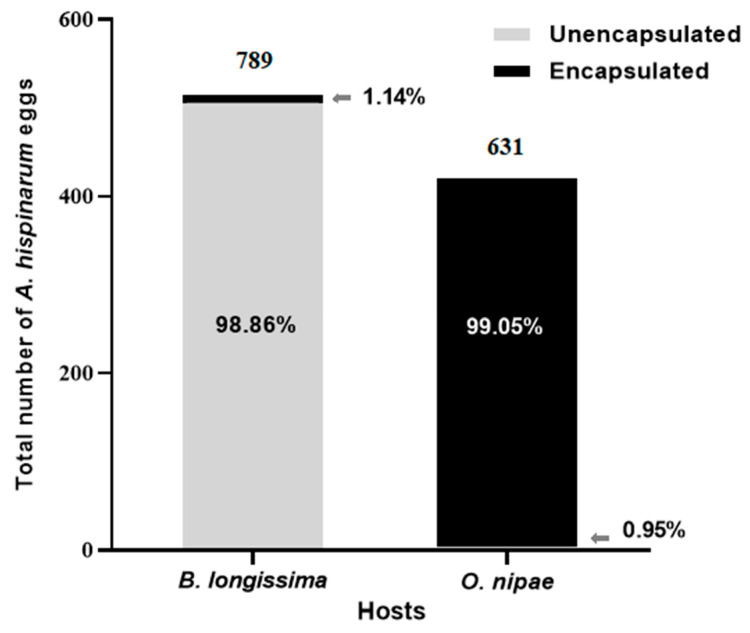
Encapsulation rate of *B. longissima* and *O. nipae* larvae on *A. hispinarum* eggs.

*O. nipae* larval hemocytes promptly encapsulated the *A. hispinarum* eggs when they were placed in *B. longissima* and *O. nipae* larvae. The encapsulation rate reached 99.05%, which was substantially greater than that of *B. longissima* larval hemocytes (1.14%) (*χ*^2^ = 1352.977, *df* = 1, *p* < 0.001) (Figure 5). Therefore, it stands to reason that eggs cannot survive in *O. nipae* larvae.

### 3.4. The Effect of the Hemocytes in B. longissima and O. nipae Larvae and the Proportion of Various Types of Hemocytes after Parasitization by A. hispinarum

#### 3.4.1. The Effect of Hemocytes in *B. longissima* and *O. nipae* Larvae after Parasitization by *A. hispinarum*

The number of hemocytes in the fourth instar larva of *B. longissima* and *O. nipae* was maintained at a largely constant level at 12, 24, 48, 72, and 96 h after feeding (Table 4). The statistics show that *O. nipae* larvae in their fourth instar have 6.08 more hemocytes than *B. longissima* larvae (*t* = −21.496, *df* = 292, *p* < 0.001).

The number of hemocytes increased considerably in *B. longissima* larvae 24 h after being parasitized by *A. hispinarum* (*t* = −3.763, *df* = 58, *p* < 0.001) (Table 4). In *O. nipae* larvae, the number of hemocytes dramatically increased 48, 72, and 96 h following *A. hispinarum* parasitism (48 h: *t* = −4.190, *df* = 58, *p* < 0.001; 72 h: *t* = −3.199, *df* = 58, *p* = 0.002; 96 h: *t* = −2.730, *df* = 58, *p* = 0.008) (Table 4). It can be seen that the change in blood cell number in *O. nipae* larvae following parasitization by *A. hispinarum* was more pronounced than it was in *B. longissima* larvae.

#### 3.4.2. The Effect of Parasitism of *A. hispinarum* on the Proportion of Various Hemocytes in Larvae of *B. longissima* and *O. nipae*

Blood cell composition and fluctuation, in addition to number, are additional indicators of the degree of encapsulation reaction. The results demonstrated that plasma cells, granular cells, oenocytoid, primitive cells, and beaded cells were the most prevalent blood cell types and morphologies in the larvae of *B. longissima* and *O. nipae*. The largest proportions were seen in plasma cells (Table 5) and granular cells (Table 6).

The proportion of plasma hemocytes in *B. longissima* larvae parasitized by *A. hispinarum* did not change significantly after 12, 24, 48, 72, and 96 h (12 h: *t* = 0.543, *df* = 58, *p* = 0.589; 24 h: *t* = −1.301, *df* = 54, *p* = 0.198; 48 h: *t* = −0.317, *df* = 58, *p* = 0.753; 72 h: *t* = 1.110, *df* = 58, *p* = 0.272; 96 h: *t* =1.728, *df* = 52, *p* = 0.090) (Table 5); the proportion of granulocytes increased and decreased significantly, at 24 and 96 h after parasitization (24 h: *t* = −2.569, *df* = 54, *p*= 0.013; 96 h: *t* = 2.552, *df* = 58, *p* = 0.013) (Table 6); the proportion of oenocytoid increased significantly at 12 h after being parasitized (*t* = −2.623, *df* = 54, *p* = 0.011) (Table 7); The proportion of primary hemocytes decreased significantly 48 h, 72 h (48 h: *t* = 3.380, *df* = 58, *p* = 0.001; 72 h: *t* = 3.866, *df* = 52, *p* < 0.001), but increased significantly 96 h after parasitism (*t* = −3.855, *df* = 58, *p* < 0.001) (Table 8).

The proportion of plasmacytes in the larvae of *O. nipae* decreased significantly at 12 h after parasitism by *A. hispinarum* (*t* = 2.043, *df* = 58, *p* = 0.046) and increased significantly at 24, 72, and 96 h (24 h: *t* = −2.479, *df* = 58, *p* = 0.016; 72 h: *t* = −2.525, *df* = 58, *p* = 0.014; 96 h: *t* = −2.419, *df* = 58, *p* = 0.019) (Table 5); the proportion of granulocytes increased significantly at 12 h after parasitization (*t* = −2.406, *df* = 58, *p* = 0.019) (Table 6); the proportion of oenocytoids gradually decreased after parasitization, and there was a significant difference at 72 h (*t* = 2.970, *df* = 58, *p* = 0.004) (Table 7); the proportion of prohemocytes increased significantly at 24 h after parasitization (*t* = 5.636, *df* = 58, *p* < 0.001) (Table 8). When compared to *B. longissima*, the changes in the proportion of hemocyte composition of the larvae after parasitization by *A. hispinarum* were more significant in *O. nipae*.

## 4. Discussion

In nature, parasitoid wasps and hosts have a balanced struggle and restraint interaction [35,36]. Researchers from all around the world have been investigating the immunological and developmental interactions between parasitoids and hosts in recent decades [37,38,39,40,41]. Depending on whether the parasitoid can develop and enclose successfully in the host, the parasitoid’s host can be classified as adaptive or non-adaptive [42]. In contrast to non-adaptive hosts, in which the host is unable to overcome the host’s immune system, adaptable hosts allow the host to suppress or avoid the immune system. During the parasitic process, the host must first contend with cellular immunity, with the host hemocytes’ envelope reaction serving as the initial hurdle [16,18].

Studies have been conducted on the selection of *A. hispinarum* as a parasite, the development interaction between *A. hispinarum* and the hosts *B. longissima* and *O. nipae*, and the hemocyte response of these hosts. The findings demonstrated that *A. hispinarum* exhibited no preference for *B. longissima* or *O. nipae* parasitic selection, but that it preferred to deposit more eggs in *B. longissima* larvae. This might be connected to how big *B. longissima* and *O. nipae* are physically. The body size of the *B. longissima* is larger than that of the *O. nipae* in all life stages, including larvae, pupa, and adults. During the parasitism process, parasitic wasps typically choose the most suitable host in which to lay eggs and tend to lay more eggs into the most suitable hosts [43]. Host density, body size, nutritional status, and developmental stage are the main influencing factors [44,45,46,47].

However, the eggs laid by *A. hispinarum* into its adaptive host *B. longissima* larvae could develop normally, whereas the eggs laid into its non-adaptive host *O. nipae* larvae were encapsulated to death and could not develop normally. In fact, in the *O. nipae* larvae, there are some *A. hispinarum* eggs that can develop into *A. hispinarum*, but this phenomenon is extremely rare; the current experiment found only one case. Non-adaptive hosts will still be affected by this process, even though parasitic wasps cannot successfully parasitize them. The parasitism of *A. hispinarum* in this experiment can cause a death rate of 15.31% of the *O. nipae* larvae and extend the larval stage by 5 days and the pupal stage by 1 day. *O. nipae* damages the young leaves of palm trees by feeding adults and larvae [48]. Field chemical control often uses beta-cypermethrin, imidacloprid, and other drugs to spray leaves [49,50]. Thus, the extension of the larval stage may allow the insect to absorb the drug more fully, thereby achieving a good control effect. Additionally, we discovered through ongoing field research that though *B. longissima* and *O. nipae* can coexist in one area, they rarely feed on the same plants. As a result, utilizing *A. hispinarum* to regulate *B. longissima* can have some negative consequences on *O. nipae*.

The encapsulation response of the host is the first thing the *A. hispinarum* eggs encounter after being deposited into the host. In this experiment, hemolymph from *B. longissima* larvae could only encapsulate 1.14% of *A. hispinarum* eggs, but hemolymph from *O. nipae* larvae could contain 99.05% of *A. hispinarum* eggs. Studies have shown a strong correlation between the overall number of host hemocytes and the number of differential hemocytes and the success of parasitic wasps [51]. Statistics show that *O. nipae* larvae had 6.08 times as many total hemocytes than *B. longissima* larvae. The amount of circulating hemocytes in the host hemolymph may have a significant role in controlling the capacity to trigger cyst response [52].

The immune system of the host is also suppressed or avoided by parasitic wasps, ensuring the normal development of the offspring [22,29,30]. The hemocytes in the *B. longissima* larvae did not react significantly to the attack by *A. hispinarum*, and only a small increase in the total number of hemocytes and an increase in the proportion of granulocytes and oenocytoids were visible in the early stages of the parasitization. This demonstrates that the cell’s immune system can act quickly, but it may be slowed down by *A. hispinarum*’s parasitic elements or rendered ineffective by the parasite’s eggs, which prevent the hemocytes from encasing the foreign substance. However, after *O. nipae* larvae were attacked by *A. hispinarum*, the total number of hemocytes grew, and the proportion of plasma and granular hemocytes increased, which formed the basis for the success of *O. nipae* larvae’s cellular immunity. According to research, the capacity of hemocytes is positively correlated with the number of circulating hemocytes in *Drosophila melanogaster* [20]. Plasmatocytes and granulocytes are key players in the encapsulation reaction, and oenocytoid cells can produce phenoloxidase to help blacken and kill bee eggs. Additionally, the original hemocytes can change into different types of hemocytes. Since the likelihood of *A. hispinarum* eggs being encapsulated by the two hosts differ significantly, it may be concluded that *A. hispinarum* eggs are capable of passive escape, which can only happen in *B. longissima* larvae.

## 5. Conclusions

The goal of this research was to look into *A. hispinarum*’s developmental interactions with *B. longissima* and *O. nipae*, as well as the hemocyte immune responses of *B. longissima* and *O. nipae* larvae to *A. hispinarum* parasitism, so that the breeding differences between *B. longissima* and *O. nipae* could be explained. The results showed that *A. hispinarum* had a preference for oviposition over the parasitism of *B. longissima* and *O. nipae*. The number of eggs laid to *B. longissima* larvae was significantly greater than that to *O. nipae* larvae, which led to a 98.95% mortality rate for *B. longissima* larvae and only a 15.31% mortality rate for *O. nipae* larvae. Another important finding is that the sufficient amount of hemocytes in *O. nipae* larvae served as the foundation for cellular immunity; the number of hemocytes in *O. nipae* larvae was 6.08 times that of *B. longissima* larvae. This study’s limitation is that it does not take humoral immunity into consideration. The fact that melanization takes place during *A. hispinarum* egg encapsulation suggests that parasitism will also cause variations in terms of how humoral immunity responds. As a result, we may further investigate various immunological effects on the two beetles from the perspective of humoral immunity.

## Figures and Tables

**Table 1:** The rate of parasitism of *A. hispinarum* on *B. longissima* and *O. nipae* and the amount of oviposition of *A. hispinarum*.

Treatment	Species	Parasitism Rate (%)	Oviposition Amount	*χ* ^2^	*t*	*df*	*p*
No-choice test	*B. longissima*	71.70 ± 1.75	-	0.032	-	1	0.859
	*O. nipae*	72.33 ± 2.36	-				
	*B. longissima*	-	18.81 ± 0.31	-	7.61	598	<0.001
	*O. nipae*	-	15.52 ± 0.30				
Dural-choice test	*B. longissima*	58.26 ± 1.55	-	0.326	-	1	0.568
	*O. nipae*	55.87 ± 1.52	-				
	*B. longissima*	-	17.31 ± 0.28	-	6.36	583	<0.001
	*O. nipae*	-	15.00 ± 0.24				

**Table 2:** Mortality of the fourth instar larvae of *B. longissimi* and *O. nipae* under unparasitization and parasitization by *A. hispinarum*.

Species	Mortality Rate (%)	
	Unparasitized	Parasitized
*B. longissima*	1.11 ± 0.33 Ba	98.88 ± 0.33 Aa
*O. nipae*	1.44 ± 0.35 Ba	15.31 ± 1.62 Ab

Note: The different uppercase letters within the same row represents significant difference between non-parasitization and parasitization by *A. hispinarum* at *p* < 0.05 level. The different lowercase letters within the same column represent significant difference between different hosts at *p* < 0.05 level.

**Table 3:** Comparison of duration of the fourth instar stage or pupae stage of *O. nipae* between non-parasitization and parasitizaiton by *A. hispinarum*.

Developmental Duration	Treatment	Days (d)	*t*	*df*	*p*
Fourth instar larvae	Unparasitized	7.17 ± 0.08	−17.244	453	<0.001
	Parasitized	12.55 ± 0.09			
Pupae	Unparasitized	8.96 ± 0.06	−44.194	577	<0.001
	Parasitized	10.64 ± 0.08			

**Table 4:** Effects of the total hemocyte counts of *B. longissima* larvae and *O. nipae* larvae parasitization by *A. hispinarum*.

Time (h)	Total Hemocyte Counts (×2.5 × 10^5^)			
	*B. longissima*		*O. nipae*	
	Unparasitized	Parasitized	Unparasitized	Parasitized
12	13.33 ± 1.33 Aa	15.30 ± 1.45 Ab	79.43 ± 9.67 Aa	86.17 ± 8.22 Ab
24	15.67 ± 2.07 Ba	28.73 ± 2.97 Aa	78.07 ± 6.87 Aa	94.60 ± 8.09 Ab
48	14.63 ± 1.94 Aa	17.50 ± 2.83 Ab	75.57 ± 8.40 Ba	125.00 ± 8.28 Aab
72	12.37 ± 1.49 Aa	15.43 ± 2.03 Ab	92.13 ± 12.71 Ba	150.43 ± 13.05 Aa
96	12.87 ± 1.56 Aa	13.90 ± 1.54 Ab	84.73 ± 12.87 Ba	132.03 ± 11.60 Aab

Note: The different uppercase letters within the same row represent significant difference between non-parasitization and parasitization by *A. hispinarum* at *p* < 0.05 level. The different lowercase letters within the same column represent significant difference between different parasitization time at *p* < 0.05 level.

**Table 5:** Effects of the percentage of plasmatocytes of *B. longissima* larvae and *O. nipae* larvae parasitized by *A. hispinarum*.

Time (h)	Percentage of Plasmatocytes (%)			
	*B. longissima*		*O. nipae*	
	Unparasitized	Parasitized	Unparasitized	Parasitized
12	38.33 ± 2.07 Ac	36.78 ± 1.97 Ab	56.49 ± 1.91 Aa	51.69 ± 1.09 Bab
24	40.72 ± 2.00 Abc	44.98 ± 2.59 Aa	49.61 ± 1.61 Babc	55.06 ± 1.50 Aa
48	47.37 ± 2.05 Aab	48.24 ± 1.79 Aa	53.03 ± 1.80 Aab	52.92 ± 1.32 Aab
72	52.81 ± 1.88 Aa	49.84 ± 1.90 Aa	43.58 ± 2.01 Bc	49.17 ± 0.99 Ab
96	54.14 ± 1.42 Aa	49.84 ± 2.04 Aa	46.70 ± 1.58 Bbc	51.67 ± 1.32 Aab

Note: The different uppercase letters within the same row represent significant difference between non-parasitization and parasitization by *A. hispinarum* at *p* < 0.05 level. The different lowercase letters within the same column represent significant difference between different parasitization time at *p* < 0.05 level.

**Table 6:** Effects of the percentage of granulocytes of *B. longissima* larvae and *O. nipae* larvae parasitized by *A. hispinarum*.

Time (h)	Percentage of Granulocytes (%)			
	*B. longissima*		*O. nipae*	
	Unparasitized	Parasitized	Unparasitized	Parasitized
12	40.24 ± 2.00 Aa	38.14 ± 2.18 Aa	27.80 ± 1.76 Bc	31.91 ± 0.86 Ab
24	35.91 ± 1.56 Ba	43.14 ± 2.19 Aa	30.90 ± 1.73 Ac	32.26 ± 0.84 Ab
48	27.72 ± 1.76 Ab	22.75 ± 2.11 Ab	33.83 ± 1.31 Abc	33.82 ± 0.92 Ab
72	24.73 ± 1.89 Ab	26.09 ± 2.10 Ab	40.62 ± 2.09 Aa	39.42 ± 0.94 Aa
96	26.87 ± 1.90 Ab	20.16 ± 1.82 Bb	38.86 ± 1.00 Aab	37.93 ± 1.22 Aa

Note: The different uppercase letters within the same row represent significant difference between non-parasitization and parasitization by *A. hispinarum* at *p* < 0.05 level. The different lowercase letters within the same column represent significant difference between different parasitization time at *p* < 0.05 level.

**Table 7:** Effects of the percentage of oenocytoids of *B. longissima* larvae and *O. nipae* larvae parasitized by *A. hispinarum*.

Time (h)	Percentage of Oenocytoids (%)			
	*B. longissima*		*O. nipae*	
	Unparasitized	Parasitized	Unparasitized	Parasitized
12	4.23 ± 0.38 Bc	5.91 ± 0.52 Ab	6.57 ± 0.79 Aa	7.37 ± 0.63 Aa
24	4.73 ± 0.53 Ac	6.49 ± 0.83 Ab	6.50 ± 0.65 Aa	6.97 ± 0.77 Aa
48	12.73 ± 1.40 Aa	15.36 ± 1.43 Aa	4.26 ± 0.42 Aa	5.16 ± 0.43 Abc
72	7.59 ± 0.71 Ab	6.44 ± 0.79 Ab	6.08 ± 0.57 Aa	4.01 ± 0.31 Bc
96	8.66 ± 0.76 Aab	11.00 ± 0.94 Aa	4.77 ± 0.56 Aa	3.54 ± 0.34 Ac

Note: The different uppercase letters within the same row represent significant difference between non-parasitization and parasitization by *A. hispinarum* at *p* < 0.05 level. The different lowercase letters within the same column represent significant difference between different parasitization time at *p* < 0.05 level.

**Table 8:** Effects of the percentage of prohemocytes of *B. longissima* larvae and *O. nipae* larvae parasitized by *A. hispinarum*.

Time (h)	Percentage of Prohemocytes (%)			
	*B. longissima*		*O. nipae*	
	Unparasitized	Parasitized	Unparasitized	Parasitized
12	6.03 ± 0.53 Ac	5.37 ± 0.38 Ac	7.80 ± 0.83 Ab	6.91 ± 0.72 Aa
24	6.30 ± 0.46 Ac	8.44 ± 0.95 Abc	10.91 ± 0.86 Aa	5.07 ± 0.56 Ba
48	8.10 ± 0.56 Abc	5.77 ± 0.40 Bb	8.35 ± 0.80 Aab	7.40 ± 0.69 Aa
72	12.83 ± 1.00 Aa	8.10 ± 0.70 Bb	6.56 ± 0.58 Ab	6.28 ± 0.59 Aa
96	10.03 ± 0.82 Bab	16.14 ± 1.43 Aa	7.15 ± 0.83 Ab	5.45 ± 0.47 Aa

Note: The different uppercase letters within the same row represent significant difference between non-parasitization and parasitization by *A. hispinarum* at *p* < 0.05 level. The different lowercase letters within the same column represent significant difference between different parasitization time at *p* < 0.05 level.

## References

[1] Maulik S. (1921). A new hispid beetle injurious to nipa palm. Ann. Mag. Nat. Hist..

[2] Waterhouse D.F., Norris K.R. (1987). Biological Control Pacific Prospects: Brontispa longissima (Gestro).

[3] Hou Y.M., Weng Z.Q. (2010). Temperature-Dependent Development and Life Table Parameters of *Octodonta nipae* (Coleoptera: Chrysomelidae). Environ. Entomol..

[4] Liu K., Fu B.L., Lin J.R., Fu Y.G., Peng Z.Q., Tang L.D. (2016). Parasitism Performance of *Tetrastichus brontispae* Ferriere over the Coconut Hispine Beetle, *Brontispa longissima* (Gestro). Neotrop. Entomol..

[5] Stapley J.H. (1980). Coconut leaf beetle (*Brontispa longissima*) in the Solomons. Alafua Agr. Bull..

[6] Chen Z.M., Wang G.H., Li M., Peng Z.Q., Ali H., Xu L.N., Geoff G.M., Hou Y.M. (2020). Development of Single Nucleotide Polymorphism (SNP) Markers for Analysis of Population Structure and Invasion Pathway in the Coconut Leaf Beetle *Brontispa longissima* (Gestro) Using Restriction Site-Associated DNA (RAD) Genotyping in Southern China. Insects.

[7] Lv B.Q., Chen Y.Q., Bao Y., Han R.D., Peng Z.Q. (2005). The feasibility of the controlling coconut leaf beetle (*Brontispa longissima*) with introducing natural enemies *Asecodes hispinarum*. Chin. Bull. Entomol..

[8] Lv B.Q., Peng Z.Q., Tang C., Wen H.B., Jin Q.A., Fu Y.G., Du Y.Z. (2005). Biological characteristics of *Asecodes hispinarum* Bouček (Hymenoptera: Eulophidae), a parasitoid of *Brontispa longissima* (Gestro) (Coleoptera: Hispidae). Acta Entomol. Sin..

[9] Minchella D.J. (1985). Host life-history variation in response to parasitism. Parasitology.

[10] Strand M.R., Pech L.L. (1995). Immunological Basis for Compatibility in Parasitoid-Host Relationships. Annu. Rev. Entomol..

[11] Poyet M., Havard S., Prevost G., Chabrerie O., Doury G., Gibert P., Eslin P. (2013). Resistance of *Drosophila suzukii* to the larvae parasitoids *Leptopilina heterotoma* and *Asobara japonica* is related to haemocyte load. Physiol. Entomol..

[12] Bitra K., Burke G.R., Strand M.R. (2016). Permissiveness of lepidopteran hosts is linked to differential expression of bracovirus genes. Virology.

[13] Li L.F., Xu Z.W., Liu N.Y., Wu G.X., Ren X.M., Zhu J.Y. (2018). Parasitism and venom of ectoparasitoid *Scleroderma guani* impairs host cellular immunity. Arch. Insect Biochem..

[14] Darsouei R., Karimi J., Dunphy G.B. (2019). Functional Characterization of Outer Membrane Proteins (OMPs) in *Xenorhabdus nematophila* and *Photorhabdus luminescens* through Insect Immune Defense Reactions. Insects.

[15] Sorrentino R.P., Carton Y., Govind S. (2002). Cellular Immune Response to Parasite Infection in the *Drosophila* Lymph Gland Is Developmentally Regulated. Dev. Biol..

[16] Parsons P., Foley E. (2016). Cellular immune defenses of *Drosophila* melanogaster. Dev. Comp. Immunol..

[17] Hillyer J.F., Strand M.R. (2014). Mosquito hemocyte-mediated immune responses. Curr. Opin. Insect Sci..

[18] Tsuzuki S., Matsumoto H., Furihata S., Ryuda M., Tanaka H., Sung E.J., Bird G.S., Zhou Y.X., Shears S.B., Hayakawa Y. (2014). Switching between humoral and cellular immune responses in *Drosophila* is guided by the cytokine GBP. Nat. Commun..

[19] McGonigle J.E., Leitão A.B., Ommeslag S., Smith S., Day J.P., Jiggins F.M. (2017). Parallel and costly changes to cellular immunity underlie the evolution of parasitoid resistance in three *Drosophila* species. PLoS Pathog..

[20] Eslin P., Prévost G. (1998). Hemocyte load and immune resistance to *Asobara tabida* are correlated in species of the *Drosophila melanogaster* subgroup. J. Insect Physiol..

[21] Fors L., Robert M., Theopold U., Hambäck P.A. (2014). Differences in cellular immune competence explain parasitoid resistance for two coleopteran species. PLoS ONE.

[22] Shi M., Chen X.X. (2015). Progress in Study on Regulation of Insect Host Physiology by Parasitoids in China. Chin. J. Biol. Control.

[23] Meng E., Qiao T., Tang B.Z., Hou Y.M., Yu W.Z., Chen Z.M. (2018). Effects of ovarian fluid, venom and egg surface characteristics of *Tetrastichus brontispae* (Hymenoptera: Eulophidae) on the immune response of *Octodonta nipae* (Coleoptera: Chrysomelidae). J. Insect Physiol..

[24] Voegele J.M. (1989). Biological control of in Western Samoa: An ecological and economic evaluation. Agr. Ecosyst. Environ..

[25] Lv B.Q., Tang C., Peng Z.Q., John L.S., Fanghao W. (2008). Biological assessment in quarantine of *Asecodes hispinarum* Bouček (Hymenoptera: Eulophidae) as an imported biological control agent of *Brontispa longissima* (Gestro) (Coleoptera: Hispidae) in Hainan, China. Chin. J. Biol. Control.

[26] Jin T., Jin Q.A., Wen H.B., Lv B.Q., Lin Y.Y., Peng Z.Q. (2012). Research Progress and Perspective Outlook of Using Parasitic Wasps to Control *Brontispa longissima*. Chin. J. Trop. Agric..

[27] He L.S., Zurian M.D., Yap M.L. (2014). A review of the status of the larvae parasitoid, *Asecodes hispinarum* Bouček, and of the pupal parasitoid, *Tetrastichus brontispae* Ferriere (Hymenoptera: Eulophidae), as biological control agents of the coconut leaf beetle, *Brontispa longissima* (Gestro) (Coleoptera: Chrysomelidae: Cassidinae), in the Asia-Pacific Region. Life Excit. Biol..

[28] Poirié M., Carton Y., Dubuffet A. (2009). Virulence strategies in parasitoid Hymenoptera as an example of adaptive diversity. Comptes Rendus Biol..

[29] Yin C.L., Li M.Z., Hu J., Lang K., Chen Q.M., Liu J.D., Guo D.H., He K., Dong Y.P., Luo J.P. (2018). The genomic features of parasitism, Polyembryony and immune evasion in the endoparasitic wasp *Macrocentrus cingulum*. BMC Genom..

[30] Kaiser M., Arvidson R., Zarivach R., Adams M.E., Libersat F. (2019). Molecular cross-talk in a unique parasitoid manipulation strategy. Insect Biochem. Mol. Biol..

[31] Ye G.Y., Hu J., Zhu J.Y., Fang Q., Yan Z.C., Wang L. (2019). Recent advances in research on the mechanisms through which parasitoid wasps regulate host immunity and development. Chin. J. Appl. Entomol..

[32] Kati A., Hardie J. (2010). Regulation of wing formation and adult development in an aphid host, *Aphis fabae*, by the parasitoid *Aphidius colemani*. J. Insect Physiol..

[33] Charles H., Godfray J. (2016). Four decades of parasitoid science. Entomol. Exp. Appl..

[34] Kim Y., Hepat R. (2016). Baculoviral p94 homologs encoded in Cotesia plutellae bracovirus suppress both immunity and development of the diamondback moth, Plutellae xylostella. Insect Sci..

[35] Aya V.M., Montoya-Lerma J., Echeverri-Rubiano C., Michaud J.P., Vargas G. (2019). Host resistance to two parasitoids (Diptera: Tachinidae) helps explain a regional outbreak of novel *Diatraea* spp. stem borers (Lepidoptera: Crambidae) in Colombia sugarcane. Biol. Control.

[36] Mathiron A.G.E., Pottier P., Goubault M. (2018). Let the most motivated win: Resource value components affect contest outcome in a parasitoid wasp. Behav. Ecol..

[37] Cai J., Ye G.Y., Hu C. (2004). Parasitism of *Pieris rapae* (Lepidoptera: Pieridae) by a pupal endoparasitold, *Pteromalus puparum* (Hymenoptera: Pteromalidae): Effects of parasitization and venom on host hemocytes. J. Insect Physiol..

[38] Gandon S., Buckling A., Decaestecker E., Day T. (2008). Host–parasite coevolution and patterns of adaptation across time and space. J. Evol. Biol..

[39] Yan Z., Qi F., Liu Y., Xiao S., Yang L., Wang F., An C., Werren J.H., Ye G.Y. (2016). A Venom Serpin Splicing Isoform of the Endoparasitoid Wasp, *Pteromalus puparum*, Suppresses Host Prophenoloxidase Cascade by Forming Complexes with Host Hemolymph Proteinases. J. Biol. Chem..

[40] Ye Z., Vollhardt I.M.G., Parth N., Rubbmark O., Traugott M. (2018). Facultative bacterial endosymbionts shape parasitoid food webs in natural host populations: A correlative analysis. J. Anim. Ecol..

[41] Cepeda A.S., Lotta-Arévalo I.A., Pinto-Osorio D., Macías-Zacipa J., Valkiūnas G., Barato P., Matta N.E. (2019). Experimental characterization of the complete life cycle of *Haemoproteus columbae*, with a description of a natural host-parasite system used to study this infection. Int. J. Parasitol..

[42] Chen Y.F. (2009). A Study on Developmental and Immunological Interactions between Parasitoids and Their Nonpermissive Host. Doctoral Dissertation.

[43] Vieira N.F., Pomari-Fernandes A., Lemes A.A.F., Vacari A.M., De B.S.A., De F.B.A. (2017). Cost of Production of *Telenomus remus* (Hymenoptera: Platygastridae) Grown in Natural and Alternative Hosts. J. Econ. Entomol..

[44] Barzman M.S., Daane K.M. (2001). Host-handling behaviours in parasitoids of the black scale: A case for ant-mediated evolution. J. Anim. Ecol..

[45] Lebreton S., Darrouzet E., Chevrier C. (2009). Could hosts considered as low quality for egg-laying be considered as high quality for host-feeding?. J. Insect Physiol..

[46] Pan M.Z., Liu T.X., Nansen C. (2018). Avoidance of parasitized host by female wasps of *Aphidius gifuensis* (Hymenoptera: Braconidae): The role of natal rearing effects and host availability. Insect Sci..

[47] Uy F.M.K., Espinoza A.M. (2018). Differential Host Handling Behavior between Feeding and Oviposition in the Parasitic Wasp *Haplogonatopus hernandezae*. J. Insect. Behav..

[48] Sun J.H., Yu P.Y., Zhang Y.Z., Wang X.J. (2003). A new invasive coconut pest in Hainan Province. Entomol. Knowl..

[49] Huang S.C., Tan W.Q., Ma Z.L., Li Z.X. (2007). Main Invasive Pests of Palm Plants in China and Their Control. Xiandai Nongye Keji.

[50] Wei Y.H., Tan W.Q., Huang S.C., Yu F.Y. (2017). Risk Analysis of *Octodonta nipae* in Hainan Province. Chin. J. Trop. Agr..

[51] Gerritsma S., de Haan A., van de Zande L., Wertheim B. (2013). Natural variation in differentiated hemocytes is related to parasitoid resistance in *Drosophila melanogaster*. J. Insect Physiol..

[52] Salt G. (1963). Experimental studies in insect parasitism-XII. The reactions of six exopterygote insects to an alien parasite. J. Insect Physiol..

